# Comparative targeting analysis of *KLF1, BCL11A,* and *HBG1/2* in CD34^+^ HSPCs by CRISPR/Cas9 for the induction of fetal hemoglobin

**DOI:** 10.1038/s41598-020-66309-x

**Published:** 2020-06-23

**Authors:** Andrés Lamsfus-Calle, Alberto Daniel-Moreno, Justin S. Antony, Thomas Epting, Lukas Heumos, Praveen Baskaran, Jakob Admard, Nicolas Casadei, Ngadhnjim Latifi, Darina M. Siegmund, Michael S. D. Kormann, Rupert Handgretinger, Markus Mezger

**Affiliations:** 1 University Children’s Hospital. Department of Pediatrics I, Hematology and Oncology, University of Tübingen, Tübingen, Germany; 2 Institute for Clinical Chemistry and Laboratory Medicine, Medical Center, Faculty of Medicine, University of Freiburg, Freiburg, Germany; 3 Quantitative Biology Center (QBiC), University of Tübingen, Tübingen, Germany; 4 Institute of Medical Genetics and Applied Genomics, University of Tübingen, Tübingen, Germany; 5 Interfaculty Institute of Biochemistry, University of Tübingen, Tübingen, Germany; 6 University Hospital Freiburg. Department of Hematology, Oncology, and Stem-Cell Transplantation, Medical Center, University of Freiburg, Freiburg, Germany; 7 University Children’s Hospital. Department of Pediatrics I, Pediatric Infectiology and Immunology, Translational Genomics and Gene Therapy in Pediatrics, University of Tübingen, Tübingen, Germany

**Keywords:** Stem-cell research, Translational research, Gene therapy

## Abstract

β-hemoglobinopathies are caused by abnormal or absent production of hemoglobin in the blood due to mutations in the *β-globin* gene (*HBB*). Imbalanced expression of adult hemoglobin (HbA) induces strong anemia in patients suffering from the disease. However, individuals with natural-occurring mutations in the *HBB* cluster or related genes, compensate this disparity through *γ-globin* expression and subsequent fetal hemoglobin (HbF) production. Several preclinical and clinical studies have been performed in order to induce HbF by knocking-down genes involved in HbF repression (*KLF1* and *BCL11A*) or disrupting the binding sites of several transcription factors in the *γ-globin* gene (*HBG1/2*). In this study, we thoroughly compared the different CRISPR/Cas9 gene-disruption strategies by gene editing analysis and assessed their safety profile by RNA-seq and GUIDE-seq. All approaches reached therapeutic levels of HbF after gene editing and showed similar gene expression to the control sample, while no significant off-targets were detected by GUIDE-seq. Likewise, all three gene editing platforms were established in the GMP-grade CliniMACS Prodigy, achieving similar outcome to preclinical devices. Based on this gene editing comparative analysis, we concluded that *BCL11A* is the most clinically relevant approach while *HBG1/2* could represent a promising alternative for the treatment of β-hemoglobinopathies.

## Introduction

Sickle cell disease (SCD) and β-thalassemia, commonly known as β-hemoglobinopathies, are inherited blood disorders caused by mutations in the human *β-globin* gene (*HBB*)^1–4^. In healthy condition, adult human hemoglobin (HbA) consists of 2 α and 2 β chains, whereas fetal hemoglobin (HbF) expressed in early gestation comprises 2 α chains and 2 γ chains. Notably, HbF was observed to bind oxygen with greater affinity than HbA, being functional when reactivated in adults^3,5,6^.

Recent studies have generated substantial experimental evidence that HbF reactivation by gene disruption of specific transcription factors and regulators could provide a therapeutic benefit for β-hemoglobinopathies^7^. It has long been appreciated that *KLF1* and *BCL11A* are key regulators involved in the process of *γ-* to *β-globin* switching and the repression of these genes leads to HbF resurgence^6–11^. Interestingly, healthy individuals with a benign genetic condition namely hereditary persistence of fetal hemoglobin (HPFH) were observed to exhibit persistent production of functional HbF^4,10,12,13^. HPFH is caused by large deletions in the *δ-* and *β-globin* genes, or point mutations in the *γ-globin* promoter and *γ-globin* repressors, such as *KLF1* and *BCL11A*^5^. Importantly, co-inheritance of HPFH with β-thalassemia was noticed to alleviate the clinical manifestations of the latter^14^. Therefore, to attain a clinical profit for β-hemoglobinopathies, several attempts were made to re-establish the expression of HbF either by lentiviral transfer of the *γ-globin* gene or by CRISPR/Cas9-mediated gene disruption of specific regulators^14–19^.

Though lentiviral gene transfer of *β-globin* exhibited positive effects in treated β-thalassemia patients^20^, the high volume of semi-random integration sites by lentivirus and the transactivation of the proto-oncogene *HMGA2* raised major safety concerns for this approach^21,22^. Due to the afore-mentioned reasons, CRISPR/Cas9-mediated gene disruption of specific regulators to re-express HbF is a promising alternative^7^. Thus, several studies have targeted various genetic regulators by CRISPR/Cas9 to reactivate HbF expression, resulting in a profound effect after genetic interference of *BCL11A*, *KLF1*, and *HBG1/2* promoters^14,17,23^. Nevertheless, no head-to-head comparison has been performed earlier in CD34^+^ hematopoietic stem and progenitor cells (HSPCs) for these three targets to assess their therapeutic potential for β-hemoglobinopathies by up-regulating HbF without raising safety issues. Therefore, in the present study, we compared all these targets in parallel for their impact on HbF resurgence and performed safety measurements by molecular analyses in order to select the best candidate for clinical translation.

## Results

### Gene editing

**Figure 1:**
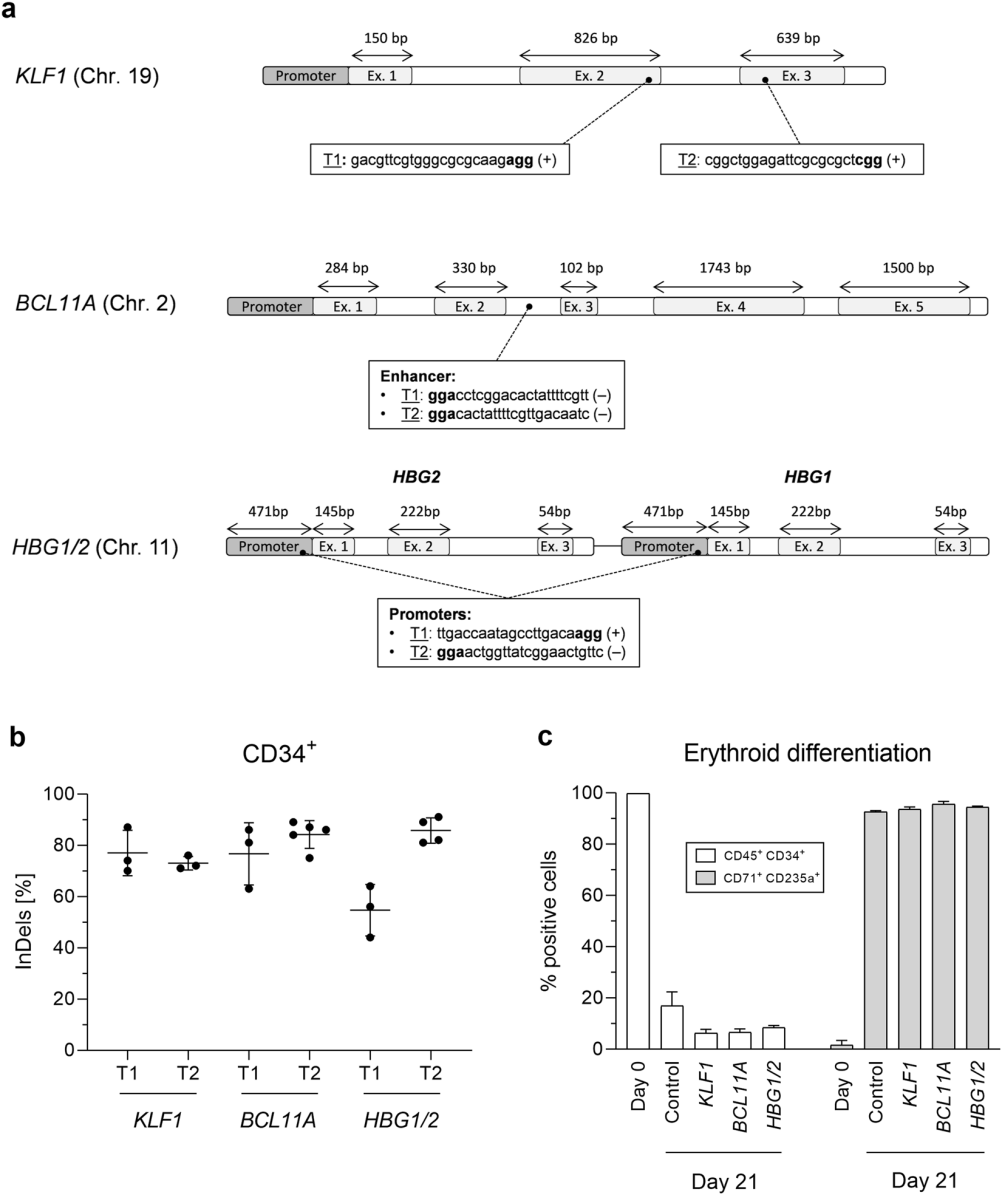
Gene editing of human CD34^+^ HSPCs. (**a**) Schematic representation of the genome-editing strategies and target sequences for each sgRNA. *KLF1*: targets 1 (T1) and 2 (T2) are localized in the second and third exon, respectively; *BCL11A*: both targets are situated in the enhancer region of the second intron; *HBG1/2*: both sgRNAs target *HBG1 and HBG2* promoters. (**b**) Indel percentage in CD34^+^ HSPCs measured by ICE analysis after electroporation of Cas9 RNP and chemically-modified sgRNAs for T1 and T2 in *KLF1*, *BCL11A*, and *HBG1/2*. (**c**) Flow cytometry analysis after immunostaining of CD34^+^ HSPCs to follow differentiation into erythrocytes precursors: percentage of double positive cells for CD34^+^ (hematopoietic stem cells) and CD45^+^ (leukocytes), and for CD71 (erythroid precursors) and CD235a (erythrocyte) on day 0 and 21, respectively.

First, we established the optimal electroporation parameters to transfect exogenous mRNA in K-562 cells and CD34^+^ HSPCs utilizing a DsRed reporter construct. Best electroporation settings were chosen for both K-562 cells (1450 V, 10 ms, 3 pulses) and CD34^+^ HSPCs (1650 V, 10 ms, 3 pulses) where high transfection efficiency and viability were achieved (>90%; Supplementary Fig. S1a). Further, to validate sgRNAs we electroporated K-562 cells with recombinant pX-330 vector targeting *KLF1*, *BCL11A*, and *HBG1/2* genomic regions. Each locus was targeted with two different sgRNAs (Fig. 1a) and gene-targeting efficacy was assessed by T7 endonuclease-I (T7E1) assay. Varying levels of mean indel frequencies were observed for *KLF1* (T1: 36.2 ± 6.5%; T2: 34.9 ± 5.1%), *BCL11A* (T1: 22.2 ± 2.2%; T2: 17.0 ± 1.4%), and *HBG1/2* (T1: 30.9 ± 14.4%; T2: 21.1 ± 6.0%; Supplementary Fig. S1b).

Next, to assess CRISPR/Cas9-mediated HbF up-regulation strategy with bone marrow-derived CD34^+^ HSPCs, we utilized Cas9 RNP with chemically modified sgRNAs instead of pX-330 vectors as the latter was shown to be less effective. We tested several molar ratios of sgRNA:Cas9 and found that a molar ratio of 2:1 was more effective in generating on-target indels (data not shown). Interestingly, elevated levels of gene editing were noticed in ICE analysis for all the tested sgRNAs (range of 63–91%; Fig. 1b), except for *HBG1/2* T1 where lower indels (54.7 ± 10.1%) were spotted. Later, gene-edited CD34^+^ HSPCs were differentiated towards erythroid lineage for 21 days, confirmed with specific erythroid markers expression (CD71 and CD235a), and molecularly analyzed for HbF expression. None of the treated samples showed proliferation or impaired erythroid differentiation (Fig. 1c).

### Transcript analysis of *γ-globin*, *KLF1* and *BCL11A*

**Figure 2:**
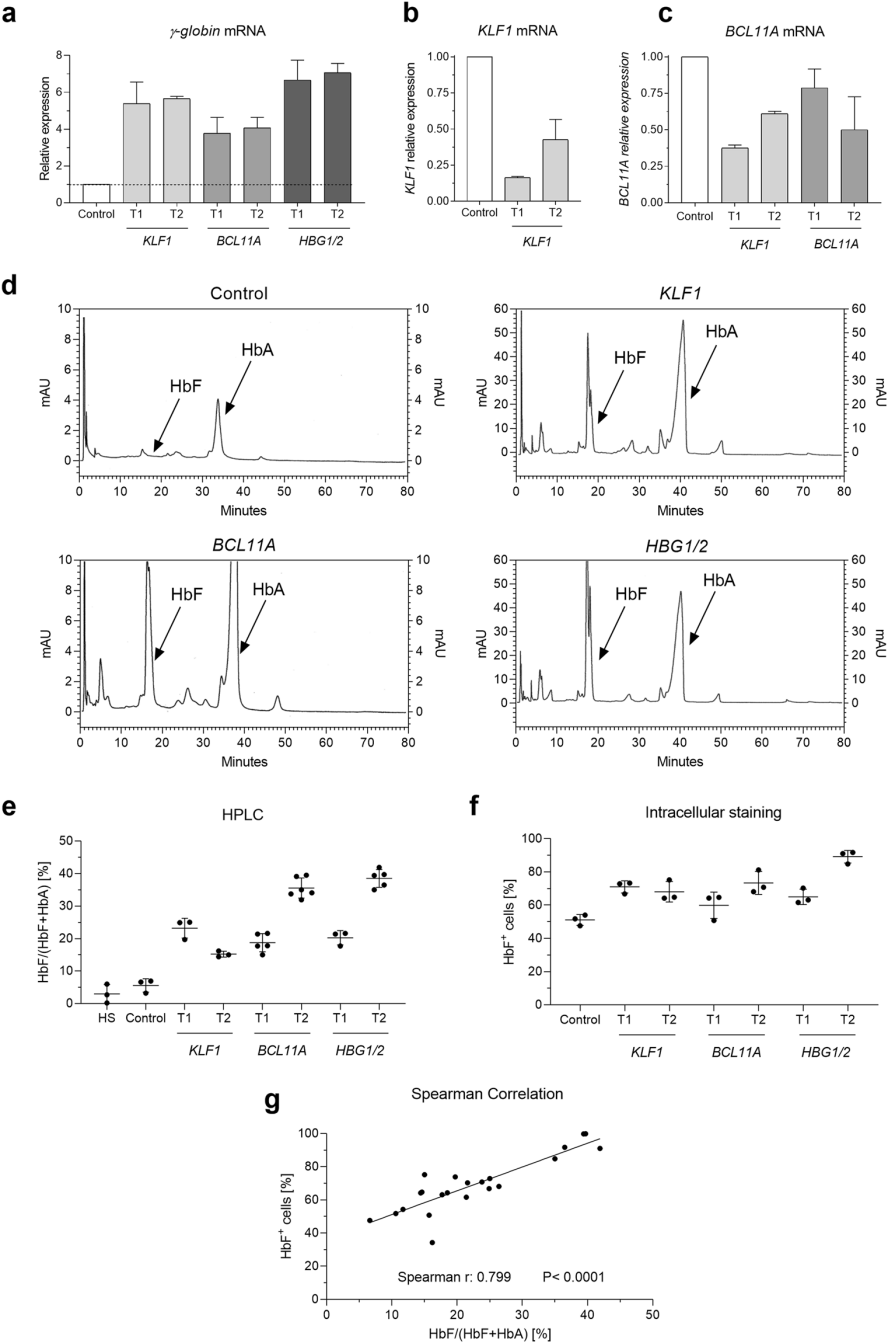
mRNA and protein analysis of gene-edited CD34^+^ HSPCs. (**a**) *γ-globin* expression analysis by qRT-PCR on day 21. (**b**) Decline of *KLF1* transcripts after treatment with *KLF1* T1 and T2. (**c**) Down-regulation of *BCL11A* transcripts in *KLF1* and *BCL11A* samples. (**d**) HPLC histograms of control, *KLF1*, *BCL11A*, and *HBG1/2* samples. (**e**) Percentage of HbF for human standard (HS), control, and the different gene editing treatments by HPLC on day 21. (**f**) HbF Intracellular staining in differentiated CD34^+^ HSPCs on day 21. (**g**) Spearman correlation of HPLC and HbF intracellular staining.

Our qRT-PCR analyses showed that, compared to control samples, HbF up-regulation was noted in *KLF1*-edited samples (>5 fold) and *BCL11A* (>4 fold) for both tested targets. Notably, elevated *γ-globin* transcripts were observed in *HBG1/2* gene-targeted samples (>6.5 fold; Fig. 2a). Also, *KLF1* and *BCL11A* transcripts were quantitatively determined by qRT-PCR, showing a marked down-regulation of KLF1 transcripts (*KLF1* T1: 4 fold, *KLF1* T2: 2 fold; Fig. 2b) with a characterized subsequent *BCL11A* down-regulation (~2 fold; Fig. 2c) after *KLF1* gene disruption. Following the same pattern, a 2-fold down-regulation of *BCL11A* transcripts was observed only in *BCL11A* T2 when the enhancer of this gene was genetically disrupted (Fig. 2c).

### HbF quantification by intracellular staining and HPLC

In order to assess HbF expression at protein level in gene-edited CD34^+^ HSPCs, cells were analyzed by HPLC-mediated hemoglobin electrophoresis and intracellular staining. Notably, hemoglobin electrophoresis revealed that all the edited samples induced higher HbF levels in comparison to the controls (Fig. 2d), while *BCL11A* T2 and *HBG1/2* T2 achieved the most pronounced HbF levels up to 39.5 and 41.9%, respectively (Fig. 2e). Moreover, *in vitro* differentiation of non-edited CD34^+^ HSPCs into erythrocyte precursors produced similar amounts of HbF as the standard human controls (Fig. 2e). After flow cytometry analysis, we found elevated numbers of HbF^+^ CD34^+^ HSPCs for all the tested target genes (range 50.8–91.7%) where the strongest effect was noted for *HBG1/2* T2 (Fig. 2f and Supplementary Fig. S1c). Of note, hemoglobin electrophoresis results strongly correlated with HbF intracellular staining (Spearman’s rho coefficient: ρ = 0.799, p < 0.0001; Fig. 2g).

### Expression pattern analysis by RNA-seq

**Figure 3:**
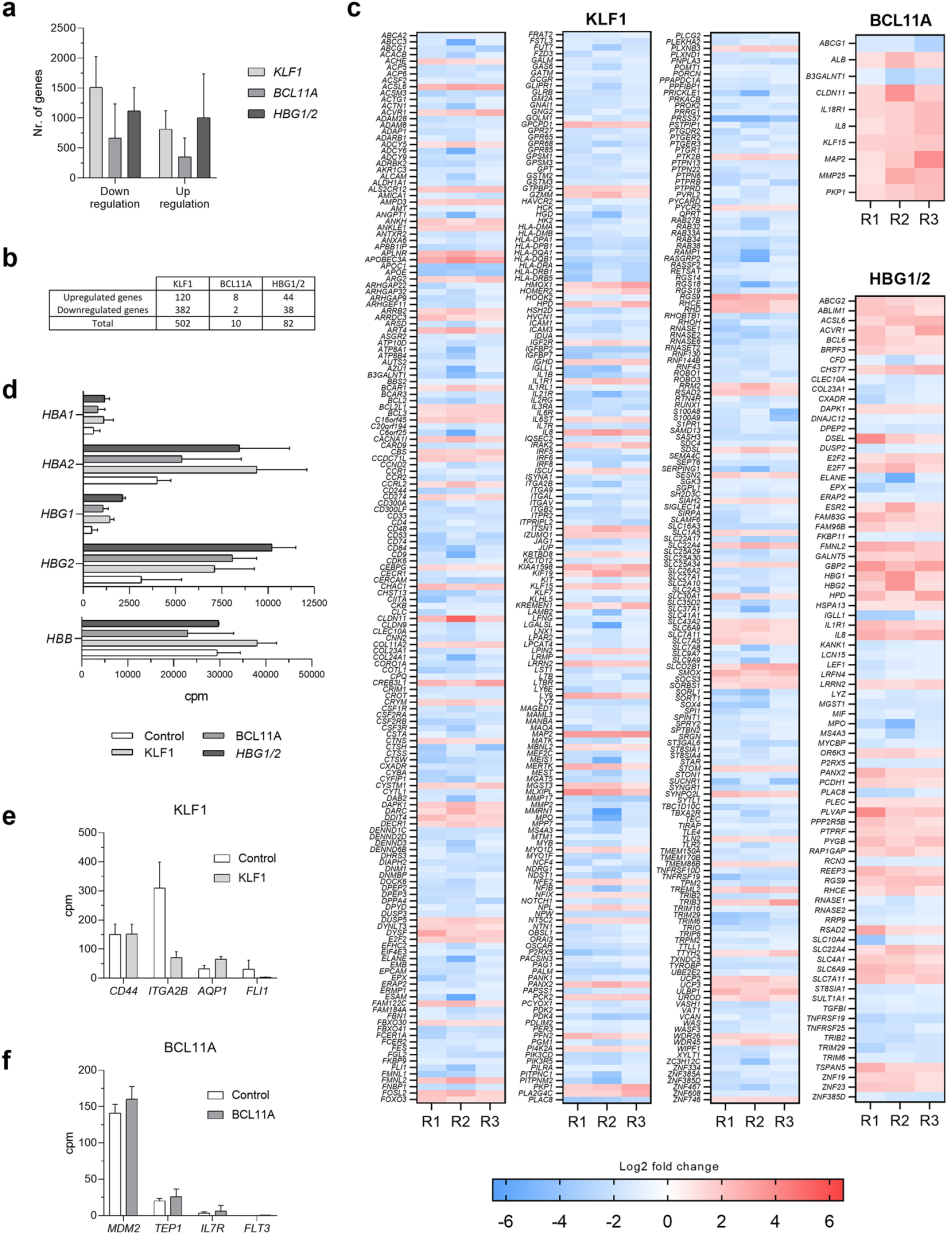
RNA-seq analysis. (**a**) Number of genes down- and up-regulated after targeting *KLF1*, *BCL11A*, and *HBG1/2*. (**b**) Number of common dysregulated genes for the three individual experiments. (**c**) Heatmap showing the Log2 fold change of the common differentially expressed genes after gene editing in *KLF1*, *BCL11A*, and *HBG1/2*. R1, R2, and R3 represent the three performed replicates. Dysregulated genes are depicted in a blue-to-red color gradient, where down-regulated genes are shown in blue and up-regulated genes in red. (**d**) Number of reads indicated as clusters per million (cpm) for hemoglobin genes in edited samples relative to the control sample. (**e**,**f**) Cpm values for the most relevant genes regulated by KLF1 (**e**) and BCL11A (**f**).

Since KLF1 and BCL11A are transcription factors involved in several signaling pathways, RNA-seq analysis was performed to determine the safety of each gene editing profile. We accounted for relatively similar ICE scores (KLF1 T1: 77 ± 8.9%; BLC11A T2: 86 ± 2.5%; HBG1/2 T2:84.7 ± 9.3%) and HbF levels (KLF1 T1: 23.2 ± 3%; BLC11A T2: 34.3 ± 0.7%; HBG1/2 T2: 39.6 ± 0.2%) to choose the samples for RNA-seq. We noticed that the expression patterns of all three gene-disrupted CD34^+^ HSPC treatments showed high similarity rates to the control sample (from 92% to 99%). Importantly, a mean value of 1017 genes showed dissimilarity in the BCL11A sample, while KLF1 and HBG1/2 gene disruption led to 2327 and 2129 impaired genes, respectively (Fig. 3a). However, when considering the common differentially expressed genes across all replicates, a clear pattern was observed, where KLF1 resulted in 502 impaired genes, whereas BCL11A and HBG1/2 accounted for 10 and 82 dysregulated genes, respectively (Fig. 3b,c). From those genes, a deeper screening for dysregulated oncogenes or tumor suppressor genes was performed to assess the safety profile of each gene editing approach. These results showed the presence of several disturbed genes involved in cell cycle (*E2F2*, *E2F7*), ERK/MAPK and p53 signaling (*DUSP2*, *PPP2R5B*, *TRIM29*), apoptosis (*DAPK1*), and immune pathways (*BCL6*) for *KLF1*- and *HBG1/2*-treated samples. In contrast, neither oncogenes nor tumor suppressor genes were found for BCL11A samples. Noteworthy, all dysregulated genes for *BCL11A*-treated samples were found in KLF1 expression panel, except for *ALB*, *IL18R*, and *MMP25*.

We also assessed the expression values indicated as clusters per million (cpm) for genes of interest involved in hematopoiesis (Fig. 3d). Predictably, adult-to-fetal hemoglobin switching was noticed in our RNA-seq results, where *HBG1/HBG2* up-regulation was noted in all treated samples. Furthermore, up-regulation of *HBA1/HBA2* was observed in those treatments where high levels of *γ-globin* transcripts were perceived. Alternatively, no major changes were detected in *HBB* expression (Fig. 3d). We also screened for important genes regulated by KLF1 and BCL11A with no dissimilarities found when compared to the control, except for *ITGA2B* (Fig. 3e,f).

### Off-target analysis by GUIDE-seq

**Figure 4:**
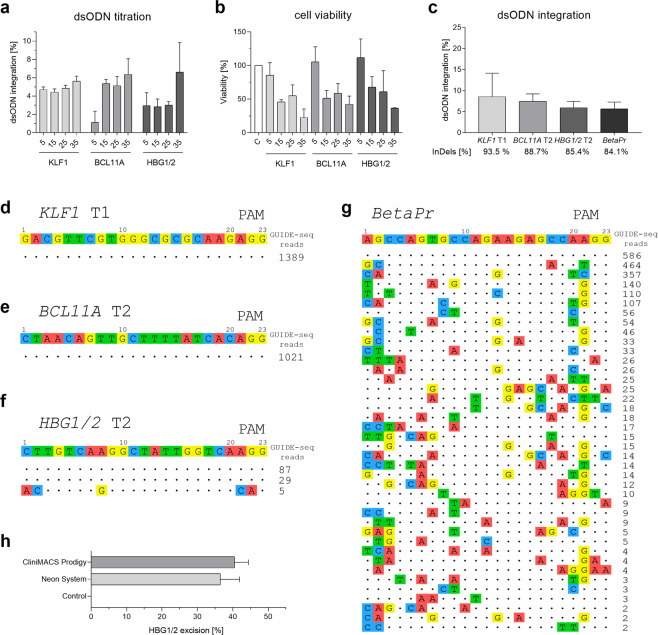
GUIDE-seq analysis. (**a**) dsODN titration in K-562 for GUIDE-seq optimization. The percentage of dsODN integration for each specific dsODN count of particle (5, 15, 25, and 35 pmol) is depicted. (**b**) Cell viability determined by cell counting on day 5 post-electroporation. (**c**) Percentage of dsODN integration at the target sites of interest utilizing 25 pmol of dsODN. Mean indel rates determined by ICE analysis are shown for each sgRNA. (**d**–**g**) Off-target illustration for *KLF1* T1 (**d**), *BCL11A* T2 (**e**), *HBG1/2* T2 (**f**), and *BetaPr* (**g**). Target sequences are shown on the top; matches are represented with dots, while mismatches are highlighted underneath. GUIDE-seq reads are shown on the right of each on-target/off-target site. (**h**) Percentage of CD34^+^ HSPCs with excised intergenic region of *HBG1/2* (5-Kb) after gene editing by Neon System and CliniMACS Prodigy.

Most importantly, GUIDE-seq analysis was performed after assessing the optimal dsODN concentration to achieve the highest integration rate with reduced cytotoxicity (Fig. 4a,b). Using 25 pmol of dsODN, we obtained adequate integration index for the most efficient targets (*KLF1* T1: 8.55 ± 5.6%, *BCL11A* T2: 7.45 ± 1.8%; *HBG1/2* T2: 5.95 ± 1.5%; Fig. 4c). We also included a sgRNA that targeted the promoter region of *HBB* (*BetaPr*) for which numerous *in silico* off-targets were predicted (Table S3, Fig. 4c). This way, our GUIDE-seq analysis resulted in no detectable off-targets, except for HBG1/2 and BetaPr, where 1 and 39 off-targets were determined (Fig. 4d–g). Interestingly, two on-targets were identified with low number of reads for *HBG1/2* T2 after GUIDE-seq (Fig. 4f). We hypothesized that, after gene editing, a 5-kb deletion between *HBG1* and *HBG2* could restrain GUIDE-seq results. With this aim in mind, we designed ddPCR oligonucleotides within the intergenic region of *HBG2* and *HBG1* (Table S2) and observed up to 43% of 5-kb excision in gene-edited samples after *HBG1/2* T2 transfection utilizing our electroporation devices (Fig. 4h).

### Translation of the gene editing platform to CliniMACS Prodigy

**Figure 5:**
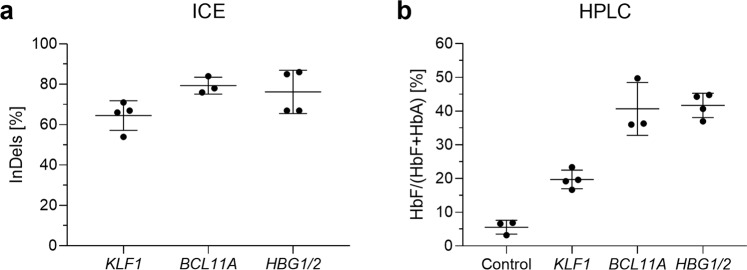
Translation of the CRISPR/Cas9 gene editing platform to the CliniMACS Prodigy GMP-grade device. (**a**) Percentage of insertions and deletions (InDels) detected by ICE analysis. (**b**) HbF levels (%) in edited human CD34^+^ HSPCs measured by HPLC.

Due to the promising outcome of all gene editing strategies, especially HBG1/2 and BCL11A, the next step was to translate this platform to the GMP-grade CliniMACS Prodigy. As previously performed in the Neon System, the best electroporation setting was selected based on DsRed mRNA transfection efficiency in CD34^+^ HSPCs, where the pulse mode ‘Square’, 600 V/100 µs first pulse, and 300 V/2 ms second pulse, was the setup combination that attained the highest percentage of DsRed^+^ cells (78.1 ± 7.2%) and cell viability (88.4 ± 13%; Supplementary Fig. S1d). Subsequently, *KLF1* T1, *BCL11A* T2 and *HBG1/2* T2 sgRNAs were transfected with the above-explained CliniMACS Prodigy settings, noticing excellent gene editing performance comparable to Neon Transfection System (range of 54–86%; Fig. 5a). Likewise, after erythroid differentiation for 21 days, HSPCs showed similar HbF resurgence by HPLC compared to Neon-transfected cells (*KLF1* T1: 19.7 ± 2.8%, *BCL11A* T2: 40.67 ± 7.8%; *HBG1/2* T2: 41.7 ± 3.6%; Fig. 5b), indicating once again the potential of these approaches for clinical translation.

## Discussion

Earlier studies relied on the fact that β-thalassemia patients with natural mutations in genetic regulators involved in the fetal-to-adult hemoglobin switching, reactivate the expression of HbF, ameliorating the disease symptoms^24^. Furthermore, mutations or epigenetic modifications in the distal promoter of *HBG1/2* can induce a similar outcome, as observed in the rare benign HPFH^14,25–28^. Above-mentioned genetic variants trigger a dynamic process, in which the HbF levels increase whereas HbA decreases. When HbF levels are above 20%, it is demonstrated to be therapeutically beneficial, especially in SCD patients due to the reduction of hemoglobin polymerization^24,26,29,30^. Therefore, owing to its great clinical impact, several investigations were attempted to induce HbF, either by gene disruption or by gene transfer^10,14,17,31^. Though gene disruption is a promising option, no comparative analyses were performed to date for these genetic loci to choose the best approach for possible clinical applications. Hence, we chose *KLF1*, *BCL11A*, and *HBG1/2* promoters as the three prominent gene regulators of ‘Globin Switching’ and performed one-to-one comparison for HbF resurgence efficacy and safety using CRISPR/Cas9-mediated gene disruption.

Several mutations have been described for *KLF1* in HPFH patients which generates variable levels of HbF (3–30.9%)^32^. In particular, our study is the first lentiviral-free attempt to use CRISPR/Cas9 to target exon 2 and 3 of *KLF1* in CD34^+^ HSPCs. We successfully induced high indel rates in both exons, down-regulation of *KLF1* and *BCL11A* transcripts, elevated *γ-globin* mRNA expression, and significant HbF levels comparable to HPFH mutations such as K288X and S270X^32,33^. Although *KLF1* gene disruption resulted in increased HbF (up to 25%) and no off-targets were detected by GUIDE-seq, the negative effect of *KLF1* knock-down was observed in our RNA-seq analysis, where several genes with different biological functions were dysregulated compared to the non-edited sample, which could raise major safety concerns. Previous studies have documented that impaired expression of *KLF1* might affect the expression of genes involved in cell-cell interaction (*CD44* and *ITGA2B*), microcytosis (*AQP1*) and cancer (*FLI-1*)^32,34^. Nevertheless, in our RNA-seq analysis no differences were perceived for those genes, except for *ITGA2B*, which its down-regulation is associated to Glanzmann thrombasthenia, a bleeding disorder characterized by a lack of platelets aggregation^35^.

Another important transcription factor implicated in *γ-* to *β-globin* switching is BCL11A^24,36–39^, which has become a promising target for HbF resurgence. Likewise, long-term engraftment and normal hematopoiesis could be attained down-regulating *BCL11A* by targeting its enhancer (GATAA box)^16,23,40^, whilst impaired erythropoiesis and limited engraftment have been detected in complete knock-down of *BCL11A* gene^23,30,36^. Therefore, in our study, we selected two sgRNAs matching the GATAA box and achieved high level of genetic disruption with excellent HbF increment up to ~40% for sgRNA T2. Due to the essential role of *BCL11A* in globin switch, regulation of proto-oncogenes (*MDM2* and *TEP1*), and genes involved in immune cell development (*IL7-R* and *FLT3*)^37,41,42^, we performed RNA-seq analyses for *BCL11A* T2 gene-edited samples. BCL11A gene disruption approach resulted in normal expression of the above-mentioned genes and led to the lowest gene expression variation of all different strategies. This low variation was expected since we are targeting the enhancer box located in the second intron of the *BCL11A* gene, and therefore, its expression declines but there is no total suppression which could affect hematopoiesis. Indeed, dysregulated genes for the BCL11A approach could also be found in KLF1 treated samples, where their expression was highly impaired since KLF1 knock-out induces stronger down-regulation of BCL11A than targeting directly its enhancer. Also, no off-targets were detected for *BCL11A* T2, hence this strategy showed high level of safety profile. In a complementary manner, a recent study performed by the group of Wu *et al*. confirmed the safety and efficacy of this sgRNA, which together with our findings could contribute in the current clinical trial for the treatment of β-thalassemia (NCT03655678) and SCD (NCT03745287)^43^.

The gamma chain of HbF is encoded by the *HBG1/2* genes and transcriptionally controlled by several elements in the *β-globin* gene cluster. Interestingly, the elevation of HbF by natural-occurring deletions in the *β-globin* cluster, varying from 13 bp, 7.2 kb (Corfu), 12.9 kb (Sicilian) and 13.6 kb, have been identified in HPFH individuals^7,14,26–28^. Furthermore, previous studies using ChIP-seq and CUT&RUN analyses elucidated the consensus binding site of BCL11A (TGACCA) repressor, situated upstream of the transcription start site of the *γ-globin* gene (−115 bp)^28,44^. Therefore, similarly to what was previously performed by Traxler *et al*., we utilized two sgRNAs to target the binding site of BCL11A, mimicking the 13-bp natural-occurring deletion described previously in HPFH individuals. This 13-bp deletion has been described before after gene editing, and might occur via microhomology-mediated end-joining (MMEJ) due to short homology sequences flanking the target site that can be observed in our ICE analysis results (Supplementary Fig. S1e)^14,28,44,45^ Notably, *HBG1/2* T2 induced higher *γ-globin* and HbF levels compared to KLF1 and BCL11A approaches. Nonetheless, RNA-seq analysis showed that HBG1/2 approach has a better safety profile than KLF1 but lower than BCL11A. After screening for possible oncogenes or tumor suppressor genes, we detected potential genes involved in oncogenesis as also observed in RNA-seq for *KLF1*-treated samples. However, to definitely confirm that those genes will not promote cancer development, gene dysregulation should also be assessed by other methods such as qRT-PCR, clonal expansion assays, and *in vivo* models. In fact, a recent publication has reported long-term engraftment of *HBG1/2*-edited HSPCs in rhesus primates and no toxic effects were found in mature blood lineages after a follow-up of 1.5 years^46^.

Since *HBG1/2* consists of two homologous subunits (*HBG1* and *HBG2*) with just some differences in the upstream region of the distal promoter, this sgRNA cleaves twice in the genome. In the present study, we confirmed that, when the sgRNA cuts simultaneously in both subunits, a 5-kb fragment is excised in high frequencies (up to 43%). Thus, the detection of the on-target reads during GUIDE-seq analysis is hindered and can only be identified when the DSB occurs either in *HBG1* or *HBG2*. These two on-targets were found in our GUIDE-seq results at low number of reads due to the above-mentioned reasons. Also, one off-target at low frequency (5 reads) was detected by GUIDE-seq for HBG1/2 sgRNA. BLAST analysis matched the off-target sequence with an intergenic region (NC_000002.12) located in chromosome 2 downstream of the ATP synthase F(0) complex subunit C3 gene and upstream of the endoplasmic reticulum junction formation factor gene. This region is associated with a long-non coding RNA (lncRNA), which might have several biological roles including epigenetic regulation^47^. This could elucidate the variation found in the transcript expression profile after gene editing, but the low number of detected reads and the uncertain function of this lncRNA lead to the inability to draw final conclusions.

To evaluate whether these approaches can be transferred to a GMP-grade electroporation device, CliniMACS Prodigy was utilized for the best sgRNAs of our study. The system is noteworthy due to its GMP-compatibility and offers automated electroporation of CRISPR components, cell culture, and direct application into humans with a clinical grade quality. Most importantly, similar results to the Neon Transfection System were attained, demonstrating the clinical potential of these gene therapy approaches. Nonetheless, *in vivo* experiments must be performed to evaluate the engraftment capacity of gene-edited cells.

Based on this thorough comparative analysis of different HbF-inducing gene editing strategies, we concluded that *KLF1* is not a suitable approach for clinical translation due to impaired gene expression after gene editing. On the contrary, *BCL11A* is a great candidate for the treatment of β-hemoglobinopathies, with high HbF resurgence, no off-targets, and unaltered gene expression. In addition, the above-explained *HBG1/2* approach also yielded clinically relevant levels of HbF with mediocre safety profile, and thus, after further investigations, this strategy could be considered a promising alternative gene therapy for β-hemoglobinopathies.

## Materials and Methods

### Ethics approval

Human mobilized peripheral blood CD34^+^ HSPCs from individual donors were adquired using protocols approved by the local ethics committee/institutional review board (IRB; ethic number: 829/2016BO2), University Children’s Hospital. Written informed consents were obtained from all the participants in the study. All methods were carried out in accordance with relevant guidelines and regulations.

### Cell culture

K-562 cells were acquired from Sigma-Aldrich and cultured at 37 °C with 5% CO_2_ in RPMI (Biochrom) supplemented with 10% FBS (Gibco), 1% L-glutamine (Biochrom), and 1% Penicillin/Streptomycin (Biochrom).

Immunomagnetic enrichment of HSPCs was performed using magnetic-activated cell sorting system (CliniMACS System, Miltenyi Biotec), according to the manufacturer’s instructions. CD34^+^ HSPCs were then cultured at 37 °C with 5% CO_2_ in StemMACS HSC Expansion Media (Miltenyi Biotec) supplemented with human cytokines (Miltenyi Biotec): SCF (100 ng/ml), TPO (20 ng/ml), and Flt3-L (100 ng/ml).

### DsRed mRNA *in vitro* synthesis

Before mRNA transcription, 20 µg pCS2^+^ DsRed was digested by XbaI (New England Biolabs) for 1 hour at 37 °C. Linearized plasmid was then purified using QIAquick PCR Purification Kit (QIAGEN) following the manufacturer’s instructions. Finally, DsRed mRNA *in vitro* transcription, poly(A) tailing, and mRNA clean up were performed in accordance with the manufacturer’s protocols of mMESSAGE mMACHINE SP6 Transcription kit (Life Technologies), Poly (A) tailing kit (Ambion), and MEGAclear kit (Ambion), respectively.

### Cloning of oligonucleotides in pX330

sgRNAs for each targeted gene were designed (Fig. 1a) and oligonucleotides cloned into the chimeric pX-330 vector (Addgene #42230; Table S1). All constructs were confirmed by Sanger sequencing. The amplification of the vectors was performed in DH5α competent cells (Sigma-Aldrich) and the purification of the plasmids by means of standard plasmid isolation kits (Peqlab Biotechnologie).

### *In vitro* differentiation of CD34^+^ HSPCs into erythrocyte precursors

CD34^+^ HSPCs were cultured according to the three-phase differentiation protocol from Dever *et al*.^48^. Subsequent erythroid differentiation and maturation were monitored by flow cytometry (BD FACSCalibur) using FITC-conjugated anti-CD34 (Miltenyi Biotec), PE-conjugated anti-CD235a (Miltenyi Biotec), PerCP-conjugated anti-CD45 (Miltenyi Biotec) and APC-conjugated anti-CD71 (Miltenyi Biotec) at two different time points, day 0 and day 21.

### K-562 and CD34^+^ HSPCs cell transfection

To transfect 1 × 10^6^ K-562 cells using the 100 µl Neon transfection kit (Thermo Fisher Scientific), 200 ng of recombinant pX-330 were utilized. Electroporation settings for this cell line were 1,450 V, 10 ms, and 3 pulses. T7 endonuclease-I (T7E1) assay was performed on day 5 after electroporation.

Chemically modified sgRNAs (Synthego; Table S1) and Cas9 ribonucleoprotein (RNP; IDT) were incubated at a molar ratio of 1:2 (45 pmol to 90 pmol) at room temperature for 15 minutes. After complex formation, 1 × 10^5^ CD34^+^ HSPCs were transfected using the 10 µl Neon transfection kit (Thermo Fisher Scientific) or the Test Cuvette Adaptor (TCA; Miltenyi Biotec) with the following electroporation settings: 1,650 V, 10 ms, 3 pulses (Neon System) or Square mode, 600 V/100 µs, 300 V/2 ms (CliniMACS Prodigy). Subsequently, cells were transferred to stem cell differentiation culture media. On day 5 post-electroporation, cells were harvested for further DNA isolation, T7E assay, and ICE analysis. On day 21, erythrocyte precursors were collected for RNA isolation, qRT-PCR, and HbF quantification. Primer sequences are listed in Table S2.

### T7E1 assay and ICE analysis

Genomic DNA was isolated 5 days post-transfection using NucleoSpin Tissue Kit following the manufacturer’s instructions (MACHERY-NAGEL). The target regions were amplified using the GoTaq Colorless Master Mix (Promega). Primers for each target region are listed in Table S2. PCR products were purified by utilizing QIAquick PCR Purification Kit (QIAGEN) and 1 μg of PCR product was used for T7E1 assay in accordance with the manufacturer’s protocol (New England Biolabs). Readouts of the assay were determined on a 2% agarose gel and analyzed by ImageJ (Fiji software). Indel rates for each target were evaluated by the web tool ‘ICE’ (Inference of CRISPR Edits; https://ice.synthego.com/) after Sanger-sequencing of the purified PCR products.

### RNA isolation, cDNA synthesis, and qRT-PCR assays

CD34^+^ HSPCs were harvested on day 21. Total RNA was isolated by using the RNeasy Mini Kit and QiaShredder spin columns (QIAGEN), in accordance with the manufacturer’s protocol. RNA at a concentration of 500 ng was used for cDNA synthesis with the QuantiTect Reverse Transcription Kit (QIAGEN). Amplification and quantification of cDNA were performed with the CFX96 Touch Real-Time PCR Detection System (Bio-Rad Laboratories). PCR was run utilizing the KAPA SYBR FAST 2x MasterMix (KAPA Biosystems). Primer sequences are listed in Table S2. Results were normalized against the expression of the housekeeping gene *β2-microglobulin* (*β2M*). The crossing point (CP) values for the unknown samples were evaluated with the formula 2 (CP *β2M* - CP target gene)^49^.

### HbF quantification

For high-performance liquid chromatography (HPLC), frozen cell pellets were lysed in 200 µl deionized sterile water and ultrasonicated for 5 minutes. Cell debris was removed by centrifugation at 13,000 g. The supernatant was then concentrated to a final volume of 30 µl using a Nanosep molecular filter (PALL Corporation) with a 10 kDa membrane by centrifugation at 13,000 g. Hemoglobin species from cell lysates were separated using a PolyCAT A cation exchanger column (PolyLC Inc). The analysis was performed on an elite-LaChrom HPLC-system (Merck-Hitachi) using a gradient elution mode with a bis-tris buffer system (buffer A: bis-tris 20 mM, NH4-acetate 13 mM, KCN 1 mM and buffer B: bis-tris 20 mM, Na-acetate 38 mM, KCN 1 mM, NaCl 200 mM). Hemoglobin proteins were detected by absorbance measurements at 415 nm. Intracellular HbF was determined 21 days after erythroid differentiation utilizing the kit ‘Monoclonal antibodies directed to HbF’ (Life Technologies).

### RNA-seq

Total RNA from edited HSPCs was isolated after 21 days of erythrocyte differentiation by RNeasy Mini Kit (QIAGEN). RNA quality was determined by measuring 260/280 and 230/260 absorbance ratios on a spectrophotometer (Nanodrop ND-1000, Peqlab Biotechnologie) and the RNA concentration using the Qubit Fluorometric Quantitation and RNA Broad-Range Assay (Thermo Fisher Scientific). The RNA Integrity Number (RIN) was determined using the Lab-on-a-Chip-System Bio-analyzer 2100 and the RNA 6000 Nano assay (Agilent).

For library preparation, mRNA fraction was enriched using polyA capture from 100 ng of total RNA using the NEBNext Poly(A) mRNA Magnetic Isolation Module (New England Biolabs). Next, mRNA libraries were prepared using the NEB Next Ultra II Directional RNA Library Prep Kit for Illumina (New England Biolabs) according to the manufacturer’s instructions. Then, the library molarity was determined by measuring the library size (approximately 400 bp) using the Bioanalyzer 2100 with the High Sensitivity DNA assay, and the library concentration (approximately 10 ng/µl) using Qubit Fluorometric Quantitation and dsDNA High sensitivity assay (Thermo Fisher Scientific). For the first experiment, the libraries were denaturized according to the manufacturer’s instructions, diluted to 1.2 pM and sequenced as paired-end 75 bp reads on an Illumina NextSeq500 (Illumina) with a sequencing depth of >22 million clusters per sample. For the second experiment, the libraries were denatured, diluted to 270 pM and sequenced as paired-end 50 bp reads on an Illumina NovaSeq6000 (Illumina) with a sequencing depth of approximately 20 million clusters per sample.

Read quality of RNA-seq data in FASTQ files was assessed using ngs-bits (v.2019_03) to identify sequencing cycles with low average quality, adaptor contamination, or repetitive sequences from PCR amplification. Reads were aligned using STAR v2.7.3a^50^ to the grch37 and the alignment quality was analyzed using ngs-bits (v.2019_11). Normalized read counts for all genes were obtained using Subread (v2.0.0) and edgeR (v3.26.8). The distribution of logarithmized cpm-normalized expression values showed similar characteristics over all samples. Based on the filtered data set, samples were investigated with respect to their pairwise similarity. Spearman’s rank correlation coefficient was calculated for each pair of samples. A hierarchical clustering was performed on the resulting similarity values. Differential gene expression analysis was conducted based on the filtered gene expression data set and a statistical model incorporating the group property of samples was tested by fitting a negative binomial distribution using a generalized linear model (GLM) approach. For the analysis, genes were classified when their gene expression fold change (log2 fold change) were equal or greater than +1, and equal or minor than −1. Only genes that were impaired in the three independent experiments were considered. Finally, a screening for oncogenes or suppressor genes was performed using Ingenuity Pathway Analysis (IPA; QIAGEN) to determine the safety of each gene therapy strategy. FASTQ files for all replicates were uploaded to Sequence Read Archive (SRA) at NCBI website (http://www.ncbi.nlm.nih.gov/bioproject/606664).

### DNA library preparation

DNA library preparation for GUIDE-seq analysis was performed as described earlier in K-562 cells^51,52^. The optimal dsODN concentration based on integration efficiency by ICE analysis and cell viability by cell counting was determined after electroporation of 100,000 cells with different dsODN concentrations (5, 15, 25, and 35 pmol). 25 pmol of dsODN was used for further transfections together with sgRNA and Cas9 RNP at molar ratio of 2:1. After 5 days in culture, DNA was isolated with DNeasy Blood & Tissue Kit using standard protocols (QIAGEN). DNA fragments of 200–450 bp were generated and subsequently ligated to adaptors by utilizing NEBNext Ultra II kit (New England Biolabs). NEBNext Ultra II Q5 Master Mix (New England Biolabs) was used for the first DNA amplification, whereas KAPA SYBR FAST 2x MasterMix (KAPA Biosystems) was utilized for the second amplification. The libraries were pooled and loaded into 3 lanes of an Illumina GAIIx single-read flow cell and two MiSeq flow cells. Bound molecules were clonally amplified on a cBot instrument. Subsequently, the first 50 nucleotides from each fragment were sequenced followed by a seven nucleotide sequencing run to decipher the barcode sequence in the adapter (Illumina).

### GUIDE-seq

Demultiplexing, PCR duplicate consolidation, cleavage site recognition, off-target activity identification, and visualization was performed with the GUIDE-Seq Analysis Package v1.0.1^53^ using the GRCh37.75 human genome as reference. The read alignment step of the pipeline was conducted using BWA-MEM v0.7.17^52^ and bedtools v2.28^54^ was used for downstream analysis.

### Droplet digital PCR (ddPCR)

PCR mastermix was prepared by adding ddPCR Multiplex Supermix (Bio-Rad Laboratories), primers (950 nM), probes (250 nM), and DNA (350 ng) at a final volume of 20 µl. Next, QX200 ddPCR droplet generator (Bio-Rad Laboratories) was utilized to separate the DNA into 20,000 droplets, which were transferred to a 96-well plate and sealed to avoid evaporation using the PX1 PCR Plate Sealer (Bio-Rad Laboratories). Finally, the PCR was run on the C1000 Touch Thermal Cycler (Bio-Rad Laboratories) with the following thermal parameters: 10 min at 95 °C, 40 cycles comprising 30 s at 95 °C, 1 min at 61 °C, and 2 min at 72 °C, followed by enzyme inactivation at 98 °C during 10 min. Finally, PCR products were examined using the QX2000 droplet reader (Bio-Rad Laboratories) and analyzed with the QuantaSoft 1.6.6 software (Bio-Rad Laboratories).

## Supplementary information

Supplementary information.
