# Supplementary material for: Comparative targeting analysis of KLF1, BCL11A, and HBG1/2 in CD34+ HSPCs by CRISPR/Cas9 for the induction of fetal hemoglobin
Source: Sci Rep. 2020 Jun 23;10:10133. doi: 10.1038/s41598-020-66309-x (PMC7311455; doi:10.1038/s41598-020-66309-x)
Supplement: Supplementary file 1 — Supplementary information. [file 41598_2020_66309_MOESM1_ESM.pdf]

SUPPLEMENTARY FIGURES AND TABLES

Comparative targeting analysis of *KLF1*, *BCL11A,* and *HBG1/2* in $CD34^+$ HSPCs by CRISPR/Cas9 for the induction of fetal hemoglobin

Andrés Lamsfus-Calle[1] *, Alberto Daniel-Moreno[1] *, Justin S Antony[1], Thomas Epting[2], Lukas Heumos[3], Praveen Baskaran[3], Jakob Admar[4], Nicolas Casadei[4], Ngadhnjim Latifi[5], Darina M. Siegmund[6], Michael S.D. Kormann[7], Rupert Handgretinger[1], and Markus Mezger[1*]

SUPPLEMENTARY FIGURES

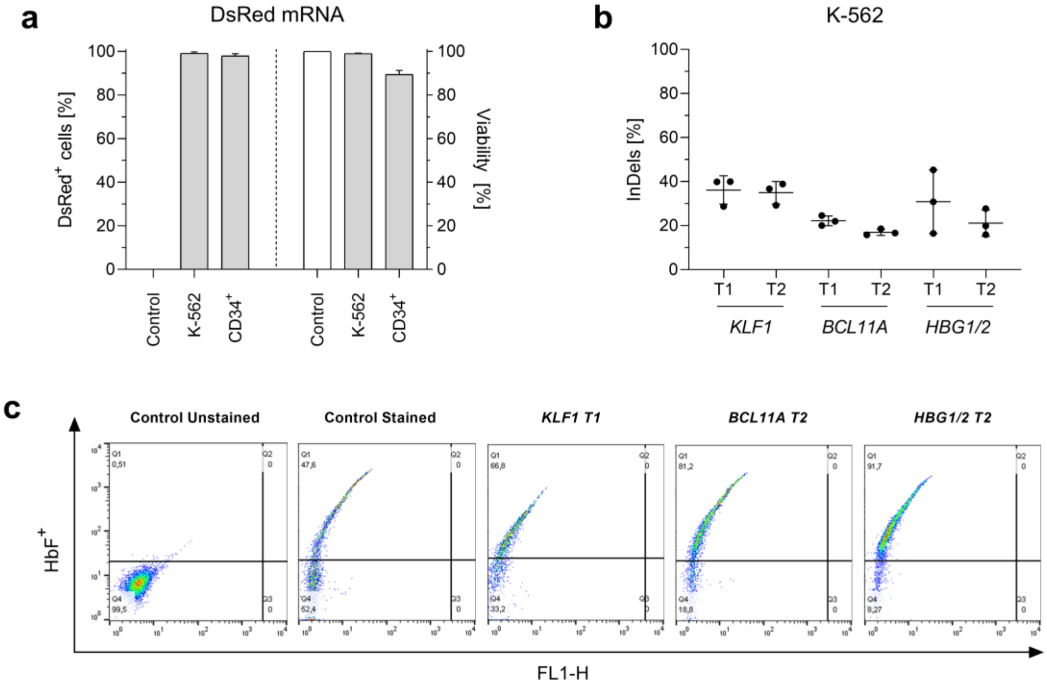

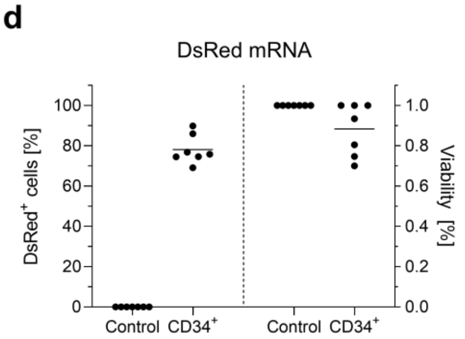

e

<table>
<tr><th>Sample</th><th>InDel [nt]</th><th>Wt sequence:
CCTTGCCTTGA|CCAATAGCCTTG</th><th>InDel[%]</th></tr>
<tr><td>1</td><td>-13</td><td>CCTTGCCT---|----------TG</td><td>50.6</td></tr>
<tr><td></td><td>-2</td><td>CCTTGCCTTGA|--AATAGCCTTG</td><td>8.2</td></tr>
<tr><td></td><td>-1</td><td>CCTTGCCTTGA|-CAATAGCCTTG</td><td>6.4</td></tr>
<tr><td></td><td></td><td></td><td>...</td></tr>
<tr><td>2</td><td>-13</td><td>CCTTGCCT---|----------TG</td><td>42.2</td></tr>
<tr><td></td><td>-2</td><td>CCTTGCCTTGA|--AATAGCCTTG</td><td>9.5</td></tr>
<tr><td></td><td>-3</td><td>CCTTGCCTTGA|---ATAGCCTTG</td><td>7.3</td></tr>
<tr><td></td><td></td><td></td><td>...</td></tr>
<tr><td>3</td><td>-13</td><td>CCTTGCC----|---------TTG</td><td>38.3</td></tr>
<tr><td></td><td>-1</td><td>CCTTGCCTTGA|-CAATAGCCTTG</td><td>3.4</td></tr>
<tr><td></td><td>-3</td><td>CCTTGCCTTG-|--AATAGCCTTG</td><td>3.1</td></tr>
<tr><td></td><td></td><td></td><td>...</td></tr>
</table>

Figure S1. Gene editing in K-562 and human CD34$^+$ HSPCs using Neon Transfection System and CliniMACS Prodigy. (**a**) Transfection efficiency and viability for K-562 and CD34$^+$ utilizing the Neon Transfection System and DsRed reporter mRNA. (**b**) Indel rates measured by T7E1-assay in K-562 cells after electroporation of recombinant pX-330 for each sgRNA. (**c**) Representative intracellular staining dot-plots showing HbF$^+$ CD34$^+$ HSPCs on day 21 for the most efficient treatments. (**d**) DsRed mRNA transfection into human CD34$^+$ HSPCs using the CliniMACS Prodigy with respective cell viability. (**e**) Representation of the 13-nt deletion detected by ICE analysis after *HBG1/2* T2 gene editing. The rectangle indicates the binding site for BCL11A which is disrupted in all gene-edited samples compared to the wild-type (wt) control sequence. Underlined are represented short homology sequences involved in MMEJ-based repair.

SUPPLEMENTARY TABLES

Table S1. Target sequences, oligonucleotides utilized for cloning into pX-330 vector, sgRNAs, and respective references for all studied genes.

Gene	Target	Target strand (5'-3')	Oligonucleotides (5'-3')	sgRNA (5'-3')	Reference
KLF1	1	**CCT** CTTGCGCGCCCACGAACGTC	For: AAAC-CTTGCGCGCCCACGAACGTC<br>Rev: CACC-GACGTTCGTGGGCGCGCAAG	GACGTTCGTGGGCGCGCAAG	17
	2	**CCG** AGCGCGCGAATCTCCAGCCG	For: AAAC-AGCGCGCGAATCTCCAGCCG<br>Rev: CACC-CGGCTGGAGATTCGCGCGCT	CGGCTGGAGATTCGCGCGCT	17
BCL11A	1	**CCT** GGAGCCTGTGATAAAAGCAA	For: AAAC-GGAGCCTGTGATAAAAGCAA<br>Rev: CACC-TTGCTTTTATCACAGGCTCC	TTGCTTTTATCACAGGCTCC	43
	2	**CCT** GTGATAAAAGCAACTGTTAG	For: AAAC-GTGATAAAAGCAACTGTTAG<br>Rev: CACC-CTAACAGTTGCTTTTATCAC	CTAACAGTTGCTTTTATCAC	43
HBG1/2	1	**CCT** TGTCAAGGCTATTGGTCAAG	For: AAAC-TGTCAAGGCTATTGGTCAAG<br>Rev: CACC-CTTGACCAATAGCCTTGACA	CTTGACCAATAGCCTTGACA	14
	2	**CCT** TGACCAATAGCCTTGACAAG	For: AAAC-TGACCAATAGCCTTGACAAG<br>Rev: CACC-CTTGTCAAGGCTATTGGTCA	CTTGTCAAGGCTATTGGTCA	14
BetaPr	1	**CCT** TGGCTCTTCTGGCACTGGCT	-	AGCCAGTGCCAGAAGAGCCA	55

Table S2. Oligonucleotide sequences utilized for PCR, qRT-PCR, and ddPCR.

Gene	qRT-PCR / PCR / ddPCR	Target	For/Rev	Primer Sequence	Reference
α-globin	qRT-PCR	-	For	CTGGCGAGTATGGTGCG	56
			Rev	GAAGTGCGGGAAGTAGGTC	56
β-globin	qRT-PCR	-	For	TGCACGTGGATCCTGAGAACT	56
			Rev	AATTCTTTGCCAAAGTGATGGG	56
γ-globin	qRT-PCR	-	For	TGGCAAGAAGGTGCTGACTTC	57
			Rev	TCACTCAGCTGGGCAAAGG	57
B2M	qRT-PCR	-	For	GATGAGTATGCCTGCCGTGT	58
			Rev	AATTCATCCAATCCAAATGAG	58
	ddPCR	Intron 1	For	TGGCTGTGATACAAAGCGGT	Own design
			Rev	GGAAACAACCAGGCAAAGAG	Own design
			HEX probe	GATGAAGAAACTAAGGCACCG	Own design
KLF1	PCR	1	For	AAGGGCACTTCCAGCTCTTC	Own design
			Rev	GTGGTCAGAGCGCGAAAAAG	Own design
		2	For	TCCTTCCTGAGTTGTTTGG	17
			Rev	GATGTCCAAACTGTCGTGCAA	Own design
	qRT-PCR	-	For	CACACAGGGGAGAAGCCATA	17
			Rev	GTCAGAGCGCGAAAAAGC	
BCL11A	PCR<br>qRT-PCR	1	For	GTGTATGTGCTGATTGAGGGC	Own design
			Rev	GGACAGCCCGACAGATGAAA	Own design
		2	For	GTGTATGTGCTGATTGAGGGC	Own design
			Rev	GGACAGCCCGACAGATGAAA	Own design
HBG1/2	PCR	*HBG1*	For	AACCACTGCTAACTGAAAGAGACT	14
			Rev	GGCGTCTGGACTAGGAGCTTATTG	
		HBG2	For	GCACTGAAACTGTTGCTTTATAGGAT	
			Rev	GGCGTCTGGACTAGGAGCTTATTG	
	ddPCR	Intergenic region	For	TTCAGTGAAGGGGGCTGAAC	Own design
			Rev	ATGACACGCTGATGCTGACT	Own design
			FAM probe	ACAGGGAGGTTGAGGTGTTA	Own design

Table S3. *In silico* off-target predictions utilizing online tool CHOPCHOP.[59]

Gene	Target	Target sequence	off-targets *in silico*
KLF1	1	GACGTTCGTGGGCGCGCAAG AGG	0
KLF1	2	CGGCTGGAGATTCGCGCGCT CGG	0
BCL11A	1	TTGCTTTTATCACAGGCTCC AGG	n/d
BCL11A	2	CTAACAGTTGCTTTTATCAC AGG	n/d
HBG1/2	1	CTTGACCAATAGCCTTGACA AGG	5
HBG1/2	2	CTTGTCAAGGCTATTGGTCA AGG	2
BetaPr	-	AGCCAGTGCCAGAAGAGCCA AGG	16

References

55. Antony, J. S. et al. Gene correction of HBB mutations in CD34+ hematopoietic stem cells using Cas9 mRNA and ssODN donors. Molecular and Cellular Pediatrics 5, https://doi.org/10.1186/s40348-018-0086-1 (2018).

56. Papapetrou, E. P. et al. Genomic safe harbors permit high β-globin transgene expression in thalassemia induced pluripotent stem cells. Nature Biotechnology 29, 73, https://doi.org/10.1038/nbt.1717 (2010).

57. Nastruzzi, C. et al. Mithramycin encapsulated in polymeric micelles by microfluidic technology as novel therapeutic protocol for beta-thalassemia. International Journal of Nanomedicine, https://doi.org/10.2147/ijn.S25657 (2012).

58. Ting, H. J., Bao, B. Y., Reeder, J. E., Messing, E. M. & Lee, Y. F. Increased expression of corepressors in aggressive androgen-independent prostate cancer cells results in loss of 1alpha,25-dihydroxyvitamin D3 responsiveness. Mol Cancer Res 5, 967–980, https://doi.org/10.1158/1541-7786.MCR-06-0318 (2007).

59 Labun, K. *et al.* CHOPCHOP v3: expanding the CRISPR web toolbox beyond genome editing. *Nucleic Acids Research* **47**, W171-W174, doi:10.1093/nar/gkz365 (2019).
